# Assessing Beliefs Underlying Rumination About Pain: Development and Validation of the Pain Metacognitions Questionnaire

**DOI:** 10.3389/fpsyg.2019.00910

**Published:** 2019-04-26

**Authors:** Robert Schütze, Clare Rees, Anne Smith, Helen Slater, Mark Catley, Peter O’Sullivan

**Affiliations:** ^1^School of Psychology, Curtin University, Perth, WA, Australia; ^2^School of Physiotherapy and Exercise Science, Curtin University, Perth, WA, Australia; ^3^School of Health Sciences, University of South Australia, Adelaide, SA, Australia

**Keywords:** metacognition, pain, assessment, catastrophizing, psychometrics, repetitive negative thinking, rasch analysis, pain management

## Abstract

Metacognitions, which are beliefs about our own thinking processes, can modulate worry and rumination and thereby influence emotional distress. This study aimed to develop a self-report measure of unhelpful pain-related metacognitions which might serve as a clinical and research tool to better understand pain catastrophizing, a significant risk factor for adverse pain outcomes. Two phases of validation are presented. Phase 1 reports on how the Pain Metacognitions Questionnaire (PMQ) was empirically developed through a qualitative study of 20 people with chronic back (*n* = 15) or knee (*n* = 5) pain in secondary or tertiary care and then validated in a large internet sample of people experiencing pain (*N* = 864). Rasch analysis yielded a 21-item scale with two dimensions (positive and negative metacognition) assessing how useful and problematic people believe rumination about pain to be, respectively. In Phase 2, further validation using a new sample (*N* = 510) replicated initial findings. Both PMQ subscales have good retest reliability (*r* = 0.76, *r* = 0.72) and internal consistency (0.86, 0.87). They correlate negatively with mindfulness and positively with pain intensity, disability, anxiety, depression, catastrophizing, rumination, and metacognition. The PMQ also predicts unique variance in catastrophizing when other variables are controlled and predicts ‘patient’ status for pain catastrophizing. Sensitivity analysis yielded preliminary suggestions for clinically meaningful cut-offs. Unhelpful pain metacognitions can be validly and reliably measured using a self-report instrument. Future studies using the PMQ might shed new light on pain-related thinking processes to develop better interventions for people prone to worry and rumination about their pain.

## Introduction

Emotional distress is a common feature of the pain experience. Prolonged distress can both be part of the sequelae of persistent pain (Banks and Kerns, 1996; Elliott et al., 2003) as well as contributing to further pain and disability, according to biopsychosocial models (Adams and Turk, 2015). For example, clinically significant symptoms of anxiety and depression are frequently comorbid with chronic pain and have been described as part of the normal psychology of pain (Eccleston, 2011) given the adverse impact pain can have on attention (Crombez et al., 2013), cognition (Eccleston et al., 2001), and behavior (O’Sullivan, 2005; Vlaeyen and Linton, 2012). Conversely, these forms of affective dysregulation can facilitate nociception through mechanisms such as central sensitization, contributing to further chronicity and a vicious cycle of pain and distress (Campbell and Edwards, 2009).

Emotional distress is therefore a common treatment target in pain management, with a large body of evidence showing psychological interventions, such as cognitive behavior therapy (CBT), can effectively reduce emotional distress (Williams et al., 2012) and even sensitization (Salomons et al., 2014). However, effect sizes are modest and unique mechanisms of change among different interventions are still poorly understood (Burns et al., 2012; Skinner et al., 2012). Even when supposed process variables that predict pain psychopathology are targeted, such as pain catastrophizing, a similar story of modest effects prevails (Schütze et al., 2018). This has prompted calls for interventions to be more theory-driven, which requires a deeper understanding of the psychological processes involved in pain-related distress (Thorn and Burns, 2011; Williams et al., 2012).

The present study aims to respond to this call by developing a tool to investigate pain-related metacognition. This is an underexplored feature of worry/rumination about pain (Schütze, 2016; Ziadni et al., 2018), which is a central aspect of perhaps the most widely studied psychological predictor of adverse pain outcomes – pain catastrophizing (PC) (Sullivan et al., 2001; Quartana et al., 2009). Metacognition is a key feature of the self-regulatory executive function (S-REF) model of psychological disorders, which suggests that prolonged emotional distress is maintained by a maladaptive strategy for dealing with unwanted thoughts, emotions and sensations (Wells and Matthews, 1994; Wells, 2000). This unhelpful coping strategy, according to the S-REF model, is termed the ‘cognitive attentional syndrome’ (CAS), which is activated and maintained by higher-order beliefs called metacognitions (Fisher and Wells, 2009). The most important of these are positive metacognitions (reflecting beliefs about how rumination can be helpful) and negative metacognitions (highlighting the harmful and uncontrollable aspects of perseverative thinking). These metacognitions can be reliably measured (Wells and Cartwright-Hatton, 2004) and modified in the treatment of various anxiety and mood disorders using metacognitive therapy (MCT) (Wells, 2009; Normann and Morina, 2018). Metacognitions are also implicitly targeted in acceptance-based and mindfulness-based interventions, which have well-established efficacy in reducing pain, disability, distress, and PC (Veehof et al., 2016; Schütze et al., 2018).

There is an emerging literature documenting associations between metacognitive beliefs and other pain outcomes, particularly PC (Yoshida et al., 2012). For example, Spada et al. (2016) found that positive meta-cognitive beliefs about worry mediated the relationship between neuroticism and PC, while negative metacognitions mediated between PC and self-reported pain behavior. Recently, Ziadni et al. (2018) further highlighted the importance of negative metacognitions in predicting greater PC and emotional distress among people with chronic pain. However, one of the acknowledged limitations of previous research is the lack of a measure of metacognition tailored for use in pain populations (Spada et al., 2016). The incumbent Metacognitions Questionnaire (Cartwright-Hatton and Wells, 1997), although widely used, was developed for a generalized anxiety disorder population. However, it only addresses worry rather than other forms of perseverative thinking, such as depressogenic rumination (Watkins, 2009), which is relevant given the frequent comorbidity of chronic pain and depression (Goesling et al., 2013). This study therefore aims to develop a psychometrically sound self-report instrument to measure positive and negative metacognitive beliefs about pain-related worry and rumination.

## Phase 1: Scale Development and Initial Validation

### Objectives

Phase 1 aimed to empirically develop items for a measure of pain-related metacognition and then test the draft scale’s psychometric properties. Aims for validation included: evaluating item functioning to discard poorly functioning items and reduce scale length; establishing construct validity (convergent and discriminant), establishing incremental utility over an existing measure; and measuring reliability (internal consistency and test–retest).

### Methods

Phase 1 was designed to empirically develop items for a measure of pain-related metacognition using a qualitative study and then validate the draft scale’s psychometric properties using a correlational study. This included: evaluating item functioning to discard poorly functioning items and reduce scale length; establishing construct validity (convergent and discriminant); and measuring reliability (internal consistency and test–retest).

#### Participants

Participants for the item generation study were 20 adults with chronic back or knee pain scoring highly on a measure of PC. This qualitative study is described in detail elsewhere (Schütze et al., 2017). Participants used for scale validation study in Phase 1 were 864 adults recruited online in two ways. The first sub-sample was a convenience internet sample of adults who responded to advertisements on websites of various pain-related organizations (e.g., Chronic Pain Australia, Australian Pain Management Association, Pain Australia, Australian Pain Society, Psoriasis and Psoriatic Arthritis Alliance), social media sites (e.g., Facebook and Twitter), and websites advertising psychological research^1^. Participants were told the research aimed to explore their beliefs about their own pain-related thoughts.

To be eligible, participants needed to be ≥18 years old, reside in a country where English is an official language, and be either experiencing an acute (non-cancer) pain episode at the time of participation or have a chronic (non-cancer) pain condition (≥3 months duration). Participants with less than moderate pain, defined as a pain intensity of less than three on a 0–10 numerical rating scale (Anderson, 2005), were screened out. Based on the total survey word count and a maximum reading speed of 450 words per minute (Carver, 1992), participants completing the survey in less than 10 min were also screened out due to high risk of not validly completing the measures. Of the 1519 participants who started the survey, 930 did not complete it, 81 did not meet inclusion criteria and two results were omitted for completing measures too quickly. This left 506 included participants in the general internet sample.

The second sample comprised a paid sample of Amazon Mechanical Turk (MTurk) workers. MTurk^2^ is an online labor market for various low-cost tasks. It has been reliably used in social science and pain research (Attridge et al., 2015), with participants shown to be more demographically diverse than the university undergraduate samples and general internet samples that are often used (Buhrmester et al., 2011; Paolacci and Chandler, 2014). Participants were given the same information about the study as the other internet sample but were each paid US$2. The same inclusion and exclusion criteria were applied. Of the 490 participants who started the survey, 70 did not complete it, 22 did not meet inclusion criteria, and 40 results were omitted for completion time of less than 10 min. This resulted in 358 included participants in the MTurk sample.

#### Measures

##### Demographics

A demographic survey was compiled based on the Electronic Persistent Pain Outcomes Collaboration patient outcomes (Blanchard et al., 2018). These included: age, gender, marital status, work status, compensation status, education level, pain onset event, pain duration, pain frequency, diagnostic status, co-morbid psychological diagnosis, acute/chronic pain status, and pain site. Demographics were used to describe characteristics of the sample, as well as to establish discriminant validity.

##### Pain metacognition questionnaire

The PMQ was developed to assess people’s unhelpful beliefs, or metacognitions, about their own pain-related thinking, which might be expected to facilitate pain rumination based on metacognitive theory (Wells, 2009). Following recommendations in scale development literature (Streiner and Norman, 2008; DeVellis, 2012), item generation was based on both theory and empirical data. This data included results from a qualitative study previously reported in detail (Schütze et al., 2017). This involved in-depth interviews about pain-related metacognitions with 15 people experiencing chronic back pain and very high catastrophizing (PCS ≥ 30). Using the same inclusion criteria, a further five people with chronic osteoarthritis knee pain who were scheduled for knee replacement surgery were also interviewed to provide perspectives from another musculoskeletal pain cohort. These interviews were based on the ‘metacognitive profiling’ interview (Wells, 2000), and also explored phenomena such as threat monitoring and stop signals. However, for the purpose of developing a scale of pain metacognitions, interpretation focused on metacognitive beliefs about worry and rumination.

As previously reported (Schütze et al., 2017), and consistent with metacognitive theory (Wells, 2009), analysis of qualitative data suggested participants’ attitudes toward perseverative thinking about pain fell into two categories: positive metacognitions (thinking about pain is helpful) and negative metacognitions (thinking about pain is unhelpful or uncontrollable). The most common positive metacognition was a belief that rumination helped participants to solve problems, while the most common negative metacognitions were that it was uncontrollable and harmful, both in terms of emotional wellbeing and pain exacerbation.

Based on these themes, metacognitive theory (Wells and Matthews, 1994; Fisher and Wells, 2009; Wells, 2009), and existing measures of metacognition (Wells and Cartwright-Hatton, 2004; Fernie et al., 2015), 40 items were drafted to allow for item reduction during scale validation. Items covered four positive metacognition themes and six negative metacognition themes, each represented with four items. These were rated on a 7-point Likert scale based on evidence that this represents an optimal number of response categories (DeVellis, 2012), ranging from ‘strongly disagree’ to ‘strongly agree.’ The draft scale was piloted on a subsample of five participants, with qualitative interviews undertaken to elicit feedback on its face validity and ease of use. Early feedback suggested that the term ‘rumination’ was not universally understood, so the terms ‘thinking a lot’ and ‘analyzing’ were used to cover perseverative thinking generally. The draft scale items are shown in Table 1.

**Table 1:** Draft 40-item version of the Pain Metacognitions Questionnaire.

Subscale	Theme	No.	Item
Positive metacognitions	Problem solving	1^∗^	My pain won’t improve unless I analyze it.
		2^∗^	When I’m thinking about pain I’m trying to problem solve.
		3	Analyzing my pain will help me to find a solution and get better.
		4	Thinking about pain doesn’t get you anywhere [R].
	Protects me	5^∗^	Thinking a lot about my pain protects me from getting injured.
		6	Thinking about my pain all the time means I’m more aware of my body so I’m less likely to hurt myself.
		7	I won’t get injured as easily if I stay focused on my pain.
		8^∗^	My pain would get worse if I didn’t think about it a lot.
	Prepares me	9	I’m better prepared for pain if I think about it a lot.
		10^∗^	Analyzing my pain prepares me for the worst.
		11^∗^	Focusing on the bad things about my pain helps me to enjoy the good things more.
		12^∗^	My pain won’t sneak up on me as long as I keep thinking about it.
	Coping	13^∗^	Thinking a lot about my pain helps me to cope with it.
		14	I should stop thinking so much about my pain because it doesn’t help [R].
		15	I feel more in control when I’m thinking about my pain.
		16^∗^	Thinking about my pain helps me to understand myself better.
Subscale	Theme	No.	Item
Negative metacognitions	Uncontrollable	17	I can’t help thinking about my pain all the time.
		18^∗^	When I start thinking about my pain, it’s impossible to stop.
		19	It’s easy to shift my attention away from thoughts about pain [R].
		20^∗^	I don’t try to stop thinking about my pain because my thoughts seem to have a life of their own.
	Increases distress	21^∗^	Thinking about my pain all the time makes me feel depressed.
		22^∗^	I’d be happier if I stopped thinking about pain.
		23^∗^	I feel stressed if I think a lot about my pain.
		24^∗^	I would be less anxious if I didn’t focus on my pain as much.
	Increases pain	25	I have less pain when I don’t think about it so much.
		26^∗^	I make my pain worse by analyzing it.
		27	It hurts more when I think about my pain too much.
		28	My thoughts don’t affect my pain levels [R].
	Social harm	29	I’m no fun to be around because I’m so focused on pain.
		30	People would like me more if I focused less on my pain.
		31	My family suffers because I think about my pain so much.
		32	If I could stop thinking about my pain, I would have better relationships.
	Must be controlled	33^∗^	I must block out my thoughts about pain.
		34	When thoughts about my pain come to mind, I try to just get on with what I’m doing [R].
		35	When thoughts about my pain grab my attention, I try to push them out of my mind.
		36^∗^	It’s important to control my thoughts about pain.
	Meta-worry	37	When I realize I’m thinking too much about my pain, I get annoyed with myself.
		38^∗^	I worry about the negative effects of thinking too much about my pain.
		39^∗^	I get caught in a vicious cycle of thinking about my pain and then thinking about how I wish I could stop thinking about it.
		40^∗^	When I find myself brooding on my pain, it starts me thinking about how I’m just making things worse.

*Items rated on 7-point Likert scale: 1 = Strongly Disagree, 2 = Disagree, 3 = Slightly disagree, 4 = Neither agree nor disagree, 5 = Slightly agree, 6 = Agree, 7 = Strongly agree. [R] indicates reverse scored items. ^∗^ Items included in final version of PMQ based on validation results.*

##### Brief pain inventory

The BPI (Cleeland and Ryan, 1994) is a 32-item instrument assessing demographic characteristics, pain intensity, medication use and functional interference. Only the 4-item pain intensity subscale and 7-item functional disability subscale of the BPI was used in the present study. Scores on these subscales range from 0 to 10, with higher scores representing more pain or disability. In cohorts of people with chronic pain, the BPI has good convergent validity and internal reliability of Cronbach’s α = 0.85 for the pain intensity subscale and α = 0.88 for the interference subscale (Tan et al., 2004). In the present sample, the pain intensity scale had a reliability of α = 0.87 while the interference scale was α = 0.93. The BPI was used to establish convergent validity.

##### Pain catastrophizing scale

The PCS (Sullivan et al., 1995) comprises 13 questions assessing the extent to which people experiencing pain report a strongly negative cognitive and affective response to pain or expected pain. The PCS has three subscales: rumination, magnification and helplessness. It has been widely validated, showing good construct validity and excellent internal consistency reliability, with α = 0.92 (Osman et al., 1997). In the present sample, it had a reliability of α = 0.93. The PCS was used to establish convergent validity.

##### Hospital anxiety and depression scale

The HADS (Zigmond and Snaith, 1983) is a 14-item measure of self-reported symptoms of anxiety and depression. It was designed for use in populations with health conditions and is not confounded by items assessing physiological symptoms of anxiety and depression like other similar measures (e.g., Beck Depression Inventory). The HADS has been widely validated and has good psychometric properties in musculoskeletal pain populations (Snaith, 2003; Pallant and Bailey, 2005). While there has been debate about the latent structure of the HADS (Coyne and Van Sonderen, 2012; Norton et al., 2012), it continues to be an effective measure of overall emotional distress (Cosco et al., 2012). In the present sample the anxiety scale had an α = 0.86 while the depression scale had an α = 0.84. The HADS was used to establish convergent validity.

##### Experience of cognitive intrusion of pain scale

The ECIP (Attridge et al., 2015) is a 10-item questionnaire measuring three aspects of cognitive intrusion in pain: intrusion, rumination, and degree of control over pain-related thinking. Initial validation showed a single factor structure and adequate construct validity in samples with no pain, acute pain and chronic pain. It has excellent internal reliability of α = 0.96 (Attridge et al., 2015). In the present study, it had a reliability of α = 0.97. The ECIP was used to establish convergent validity.

##### Tampa scale of kinesiophobia

The TSK (Miller et al., 1991) is a 17-item instrument measuring fear of movement, pain and injury. It was originally developed for use with CLBP patients (Vlaeyen et al., 1995) and has been validated in other populations of people with musculoskeletal pain (Roelofs et al., 2007), as well as heterogeneous chronic pain samples (Cook et al., 2006). The TSK has good construct validity and internal consistency reliability ranging from adequate to good (α = 0.76 to α = 0.84) (Crombez et al., 1999; French et al., 2007). It had a reliability of α = 0.85 in the present study. The TSK was used to establish convergent validity.

##### Metacognitions questionnaire

The MCQ-30 (Wells and Cartwright-Hatton, 2004), a shortened version of the original MCQ (Cartwright-Hatton and Wells, 1997), is a 30-item measure of metacognitive beliefs associated with worry and rumination. Higher scores on the MCQ are positively associated with obsessive-compulsive symptoms, pathological worry, and depression, amongst other symptoms. It has a five-factor structure (positive metacognitive beliefs, cognitive confidence, cognitive self-consciousness, uncontrollability/danger, and need for control) that correlates well with measures of worry and anxiety, thereby demonstrating good construct validity. The MCQ-30 has excellent internal consistency reliability (α = 0.93) and good test–retest stability (α = 0.75) (Wells and Cartwright-Hatton, 2004). Internal consistency was α = 0.91 in the present study. The MCQ-30 was used to establish convergent validity. Although another metacognitive measure has been used in at least one pain population (Fernie et al., 2015; Kollmann et al., 2016), the MCQ was chosen as a validation measure because it is much more widely used and validated.

##### Mindful attention awareness scale

The MAAS (Brown and Ryan, 2003) is a 15-item instrument measuring people’s self-reported moment-to-moment awareness of their actions, thoughts, sensations, emotions, and interpersonal interactions. It has good convergent and discriminant validity, excellent test–retest reliability (*r* = 0.81), and good internal consistency, with α = 0.87 (Brown and Ryan, 2003). The MAAS has been found to correlate negatively with pain catastrophizing (Schütze et al., 2010; Day et al., 2014). In the present study, internal consistency of MAAS was α = 0.93. The MAAS was used to establish convergent validity.

##### Patient global impression of change

The PGIC is a single item scale of a patient’s overall evaluation of how much their condition has improved after a treatment (Guy, 1976). The version used in this study is rated on a 7-point scale from “no change” to “a great deal better, and a considerable improvement that has made all the difference” (Hurst and Bolton, 2004). Its construct validity in pain samples has been established through strong associations with pain intensity (Farrar et al., 2001) and other outcomes such that it is recommend as a core outcome measure of overall improvement with treatment in pain trials, especially in interpreting clinically significant change (Dworkin et al., 2008). The PGIC was used to examine whether changes in PMQ scores in the test–retest analysis were related to changes in pain status.

#### Procedure

This study received ethical approval from the Government of Western Australia, Department of Health (SMHS 2014-079) and Curtin University (HR23/2015). Participants responding to online advertisements described above were directed to a study link within the Qualtrics (Provo, UT, United States) online platform, which contained participant information, informed consent questions, inclusion criteria screening questions, and the measures described above. Each measure was presented on a separate webpage and participants were required to answer all questions to progress through the survey and have their responses included. Participants were also asked for an email address to enter the reward draw and be sent a link to the PMQ retest survey 1 week after initial assessment. This automated email was sent to the first 200 participants, since this was deemed adequate to power the test–retest correlations. Using G^∗^Power, we calculated that a sample of 191 was required to have an 80% chance of detecting a weak (*r* = 0.2) correlation (Faul et al., 2007). The retest survey only included the PMQ and the one-item PGIC. Participants were free to withdraw at any time.

#### Analysis

The analytic strategy was to firstly assess item performance and remove poorly functioning items to refine the scale. Re-analysis of the shortened scale was then planned to report item functioning, scale properties and construct validity.

##### Rasch analysis of item functioning

Psychometric properties of the draft 40-item scale were evaluated with Rasch analysis using Winsteps software (v4.0.0^3^). Rasch analysis is based on item response theory rather than classical test theory and has several advantages, including producing true interval-level scales rather than ordinal ones (Pallant and Tennant, 2007; DeVellis, 2012). Tennant and Conaghan (2007) provide a useful overview of the Rasch model. The Andrich Rating Scale model was indicated and supported since the Likert-type scale categories are shared across items and no meaningful improvements in item and person statistics were noted when the Partial Credit model was applied (Wright, 1998). The following components were evaluated: dimensionality, targeting, item and person fit, category ordering, internal consistency and differential item functioning.

An exploratory analysis was conducted to assess the suitability of the sample and the unidimensionality of the draft scale items. The suitability of the data for scale evaluation was assessed by comparing how well the scale items targeted the sample. Item endorsability (i.e., how easy an item was to endorse) and person agreeability (i.e., how agreeable the sample were) was assessed by visual inspection of the person-item distribution map and comparison of summary statistics. An exploratory principal components analysis of residuals (PCA) was conducted to determine whether the 40-item scale constituted a unidimensional measure of pain metacognitions or a bi-dimensional measure of positive and negative metacognitions. The residual correlation matrix was visually inspected to identify item clusters with substantial loadings (eigenvalue greater than 2). The outcome of the exploratory PCA determined whether the scale was further considered in its entirety or as distinct subscales of related but independent constructs.

The item and person fit statistics were compared to identify items that functioned poorly. Fit statistics are chi-square–based and are reported as mean squares (in logits) with an expected value of 1 logit. The characteristic curves of items with infit (information-weighted) and outfit (outlier-sensitive) fit statistics >1.3 (model underfit) or <0.7 (model overfit) were analyzed. Poor person fit due to unexpected response patterns may compromise item fit. Such response patterns may reflect responder carelessness; hence, for the purpose of scale calibration, people with infit or outfit statistics >2 or <0.3 logits were removed prior to reanalysis (Tennant and Conaghan, 2007).

Local dependence of items infers that the response on one item determines the response on another and can inflate reliability (Tennant and Conaghan, 2007). Items with residuals that correlated strongly were reviewed to determine whether they duplicate each other, and are thus redundant, or contribute to multidimensionality. To assess the function of the Likert-type scale categories, the category ordering was assessed. Seven response categories (1–7) were proposed, thus each item had six step-calibrations – the thresholds at which the likelihood of endorsing one category is equal to that of endorsing the next. Disordered step-calibrations are indicative of under-utilized categories and can influence the function of the scale. If disordering was detected, the Likert-type scale categories were collapsed to explore whether fewer categories improved the fit of the items.

Internal consistency reliability was assessed using the Person Separation Index (PSI) as an indicator of how reliably the scale differentiates people of differing agreeability. A PSI value >0.8 in Winsteps implies the scale is sensitive enough to distinguish between individuals with high and low agreeability, suggesting good reliability.

An analysis of Differential Item Functioning (DIF) detects whether participant attributes bias the responses to items, contributing to item misfit. DIF was conducted to assess the influence of gender (male and female) and six further characteristics, each dichotomized according to sample median: age (younger ≤ 38 years, older > 38 years), pain duration (shorter ≤ 5.25 years, longer > 5.25 years), pain intensity (low BPI-P ≤ 5, high BPI-P > 5), pain interference (low BPI-I ≤ 5.3, high BPI-I > 5.3), pain catastrophizing (low PCS ≤ 20.5, high PCS > 20.5), pain cognitive intrusion (low ECIP ≤ 26, high ECIP > 26), psychological distress (low HADS ≤ 16, high HADS > 16). Statistically significant (*p* < 0.01) contrasts > 0.5 logits were deemed indicative of bias.

Items that exhibited excessive fit statistics or demonstrated local dependence were reviewed and considered for removal. The remaining items were then re-analyzed with the people deemed to be misfitting excluded.

##### Temporal stability

Temporal stability of the PMQ was assessed by correlating scores on the PMQ at two times, 1 week apart, with correlations of *r* ≥ 0.70 providing evidence of good test–retest reliability (DeVellis, 2012).

##### Construct validity

Construct validity was evaluated in terms of convergent and discriminant validity. Evidence of convergent validity included significant positive correlations between the PMQ and conceptually related pain outcomes (BPI, PCS, TSK, and ECIP), psychological distress (HADS), and metacognition (MCQ-30), as well as significant negative correlations with mindfulness (MAAS). Evidence of discriminant validity was sought in the form of non-significant correlations between the PMQ and demographic variables having no expected *a priori* relationship: country of residence, marital status and education level. Pearson product moment correlations were calculated in IBM SPSS for Macintosh version 24.0 (IBM Corp, 2016). To test whether the PMQ performs better than the existing MCQ-30, multiple hierarchical regression was also used. Using pain catastrophizing (PCS) as the dependent variable, the MCQ-30 subscales were entered as predictors in the first block, and the two PMQ scales were entered in the second block to test whether the PMQ explains unique variance in the PCS once the MCQ-30 is controlled.

### Results

#### Sample Characteristics

The sample (*N* = 864) was represented by 34 different countries; however, most participants lived in the United States (*n* = 364, 42.1%), United Kingdom (*n* = 193, 22.3%), Australia (*n* = 223, 25.8%) or Canada (*n* = 20, 2.3%). Demographic characteristics of the combined sample are shown in Supplementary Table S1. Most were female (*n* = 585, 67.7%) and the mean age was 39.7 years (*SD* = 12.6). The mean pain duration was 8.40 years (*SD* = 8.93). A large proportion were employed (*n* = 542, 62.7%) and most were not involved in compensation claims (*n* = 803, 92.9%). Almost all participants identified as living with chronic pain (*n* = 814, 94.3%) and experiencing a pain episode at the time of assessment (*n* = 854, 98.8%). Most (*n* = 508, 58.8%) reported having been given a diagnosis for their pain condition, while around a quarter (*n* = 215, 24.9%) reported having a comorbid mental health diagnosis from a health professional.

Pain sites experienced by the sample are detailed in Supplementary Table S2, showing that low back pain was most common (*n* = 347, 40.2%). Average scores of the sample on the pain and psychological outcomes described above are presented in Table 2. This shows that on average the sample had moderate pain (Anderson, 2005), high catastrophizing that exceeded a conservative risk threshold of 20 on the PCS (Sullivan et al., 2006; Wideman et al., 2009), high fear based on a TSK cut-off of 40 (Wertli et al., 2014), high anxiety based on a clinical cut-off of 8 (Bjelland et al., 2002), and sub-clinical depressive symptoms based on the same cut-off. Within the subsample of 172 participants completing the PGIC 1 week after initial assessment, the median overall symptom change was 2 (“almost the same”) with an interquartile range of 2 to 4 (“somewhat better”). Only 6 participants (3%) registered a clinically significant score of 6 (“better”) or 7 (“a great deal better”). This suggests participants’ main symptoms in the re-test sample did not change significantly between the two assessment points.

**Table 2:** Scores on pain and psychological outcome measures for initial validation sample (*N* = 864).

Outcome	Mean	*SD*	Range	Interpretation
Positive pain metacognitions (PMQ-P)	28.26	9.79	9-61	–
Negative pain metacognitions (PMQ-N)	49.32	11.94	12-79	–
Pain intensity (BPI-P)	4.89	1.66	0.25-10	Moderate (Anderson, 2005)
Pain interference (BPI-I)	5.07	2.47	0-10	–
Pain Catastrophizing (PCS)	21.05	10.75	0-52	High (Scott et al., 2014)
Cognitive intrusion of pain (ECIP)	25.94	15.33	0-60	–
Fear of pain (TSK)	39.69	7.61	18-63	High (Wertli et al., 2014)
Depression (HADS-D)	6.93	4.29	0-20	Sub-clinical (Bjelland et al., 2002)
Anxiety (HADS-A)	8.92	4.66	0-21	Clinical (Bjelland et al., 2002)
Metacognition (MCQ)	60.95	15.05	31-115	–
Mindfulness (MAAS)	3.83	0.93	1.07-6	–

*SD, standard deviation.*

#### Rasch Analysis of Item Functioning

**FIGURE 1:**
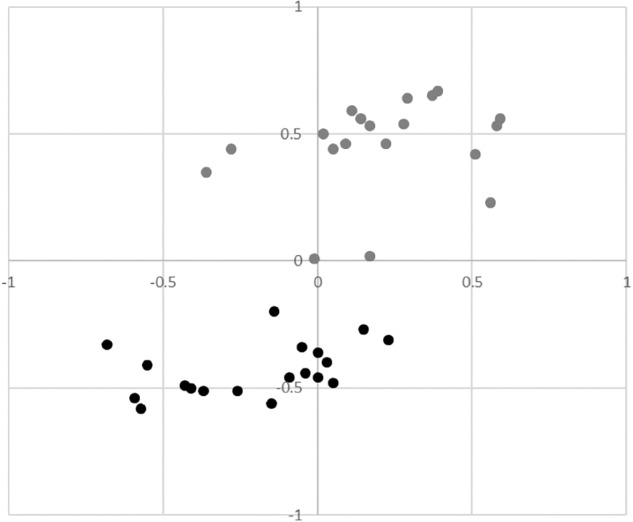
Principal Components Analysis residual correlation matrix showing the distinction between positive metacognitions (grey) and negative metacognitions (black).

Rasch analysis was conducted using the data from 864 people. The exploratory analysis suggested the sample was suitable for analysis: a mean (SD) person agreeability of -0.25 (0.37) logits was comparable to the default mean item endorsability of 0.00 (0.33) logits and visual inspection of the person-item map demonstrated an even distribution. Visual analysis of the PCA residual correlation matrix, however, clearly demonstrated a distinction between positive and negative metacognitions as independent constructs (see Figure 1). A contrast eigenvalue of 8.3 supported the finding of multidimensionality and the positive and negative item subscales were thus considered separately.

Each subscale targeted the sample reasonably well. For the positive subscale, the mean (SD) person agreeability was -0.64 (0.82) logits (range = -4.54 to 4.00 logits) lower than the default mean (SD) item endorsability of 0 (0.38) logits (range = -0.81 to 0.56 logits), suggesting the items targeted were relatively hard to endorse. The negative subscale items better targeted the sample with a mean person agreeability of -0.12 (0.51) logits (range = -3.08 to 2.04 logits) comparable with the mean item endorsability of 0 (0.34) logits (range = -0.62 to 0.80 logits). Supplementary Table S3 shows the average item endorsability thresholds in hierarchal order where higher item thresholds indicate harder to endorse items. Seven people (<1%) scored a minimum score on the positive subscale and no one scored an extreme score on the negative subscale, suggesting floor and ceiling effects are negligible.

Supplementary Table S3 also summarizes the fit statistics for the two subscales. Items 2, 4, and 14 of the positive subscale were shown to underfit the model; items 2 and 14 demonstrated excessive infit and items 2, 4, and 14 demonstrated excessive outfit. Items 25, 28, and 34 of the negative subscale were shown to underfit the model; items 25 and 28 demonstrated excessive infit and items 25, 28, and 34 demonstrated excessive outfit. Misfitting items were further explored and considered for removal.

Seventy-nine people demonstrated excessive underfit and 37 demonstrated excessive overfit for the positive subscale. Sixty-five people demonstrated excessive underfit and 26 demonstrated excessive overfit on the negative subscale. Visual inspection of the PCA correlation matrices suggested items 1, 2, 3, and 4 of the positive subscale and items 25, 26, 27, and 28 of the negative subscale potentially constitute secondary dimensions. Eigenvalues of 3.2 and 4.1, respectively, supported this notion. Assessment of local dependence revealed meaningful relationships between items 1, 2, and 3, items 5, 6, and 7, items 9 and 10 and items 15 and 16 of the positive subscale. Relationships between items 17 and 18, items 25, 26, and 17 and items 37 and 38 of the negative subscale were noted also. Correlated items were inspected and items deemed redundant were removed.

Visual inspection of the category ordering showed categories 3 and 4 of both subscales were disordered suggesting they were underutilized by the sample. The disordering was resolved by collapsing categories 2 and 3 and categories 4, 5, and 6, suggesting a 4-point Likert scale may function better. The positive and negative subscales were shown to be reliable, with person separation indices of 0.91 and 0.90, respectively. However, reliability may have been inflated due to local dependence between several subscale items.

No significant differences in item functioning were observed for gender, age, pain duration or pain intensity in either subscale. Items 4 and 14 of the positive subscale were notably harder to endorse (0.37 and 0.38 logits, respectively) by people with higher levels of PC and item 14 was notably harder to endorse (0.35 logits) by people with higher levels of pain cognitive intrusion. Item 25 of the negative subscale was meaningfully biased by pain interference (0.51 logits), pain cognitive intrusion (0.57 logits), psychological distress (0.52 logits) and PC (0.62 logits), being notably harder to endorse for people with higher levels of each characteristic. Item 17 was also notably harder (0.50 logits) to endorse by people with higher levels of pain cognitive intrusion and item 28 was notably harder (0.52 logits) to endorse by people with higher levels of PC. These biases likely contributed to the misfit of these items.

Reanalysis of the positive and negative subscales was conducted on data for 814 and 809 people, respectively, after misfitting people were excluded. Seven positive (items 3, 4, 6, 7, 9, 14, and 15) and seven negative (items 19, 25, 27, 28, 34, 35, and 37) subscale items were excluded because they functioned poorly or were deemed redundant. Four additional items related to social factors (29, 30, 31, and 32) were removed because responses to these items may reflect the impact *pain* has on people’s relationships rather than the impact *thinking about* pain has on relationships. While item 2 showed misfit and local dependence with item 1, it was considered important given the prominence of the problem solving theme in the qualitative scale development study (Schütze et al., 2017), and it was therefore retained.

Reanalysis supported the refining of the subscales. Table 3 shows the average item endorsability thresholds and fit statistics for the refined subscales. Targeting of the positive subscale was improved; mean person agreeability 0.42 logits (range = -4.97 to 4.94 logits) compared to mean item endorsability of 0 logits (range = -1.71 to.96 logits). Targeting of the negative subscale was comparable; mean person agreeability 0.01 logits (range = -2.18 to 2.84 logits) compared to mean item endorsability 0 logits (range = -0.65 to 0.61 logits). Twelve people (1.5%) registered a minimum score on the positive subscale and 1 person (<1%) registered a maximum score. No people scored an extreme score on the negative subscale, suggesting floor and ceiling effects are negligible for both subscales.

**Table 3:** Categorical order, item endorsability thresholds and fit statistics for the revised 21-item version of the Pain Metacognitions Questionnaire.

Positive Metacognitions Subscale (*N* = 814)						
**Item**		**Threshold**	**Score**	***SE***	**Infit**	**Outfit**

12	My pain won’t sneak up on me as long as I keep thinking about it.	0.96	2013	0.06	0.76	0.73
8	My pain would get worse if I didn’t think about it a lot.	0.89	2050	0.06	0.93	0.95
11	Focusing on the bad things about my pain helps me to enjoy the good things more.	0.78	2152	0.06	1.02	1.02
13	Thinking a lot about my pain helps me to cope with it.	0.47	2308	0.06	0.75	0.75
5	Thinking a lot about my pain protects me from getting injured.	-0.21	2720	0.06	1.14	1.14
10	Analyzing my pain prepares me for the worst.	-0.29	2783	0.06	0.84	0.83
16	Thinking about my pain helps me to understand myself better.	-0.35	2816	0.06	0.89	0.89
1	My pain won’t improve unless I analyze it.	-0.56	2909	0.06	1.25	1.26
2	When I’m thinking about my pain I’m trying to problem solve it.	-1.71	3591	0.06	1.42	1.40
**Negative Metacognitions Subscale (*N* = 809)**					
20	I don’t try to stop thinking about my pain because my thoughts seem to have a life of their own.	0.61	2655	0.03	1.36	1.51
18	When I start thinking about my pain, it’s impossible to stop.	0.53	2750	0.03	1.14	1.18
26	I make my pain worse by analyzing it.	0.43	2858	0.03	1.12	1.15
39	I get caught in a vicious cycle of thinking about my pain and then thinking about how I wish I could stop thinking about it.	0.35	2959	0.03	0.83	0.83
33	I must block out my thoughts about pain.	0.22	3111	0.03	0.97	1.00
40	When I find myself brooding on my pain, it starts me thinking about how I’m just making things worse.	0.15	3188	0.03	0.71	0.72
38	I worry about the negative effects of thinking too much about my pain.	-0.06	3425	0.03	0.96	0.98
24	I would be less anxious if I didn’t focus on my pain as much.	-0.26	3641	0.03	0.88	0.91
21	Thinking about my pain all the time makes me feel depressed.	-0.3	3689	0.03	1.13	1.11
22	I’d be happier if I stopped thinking about pain.	-0.49	3884	0.03	0.84	0.85
23	I feel stressed if I think a lot about my pain.	-0.52	3913	0.03	0.94	0.89
36	It’s important to control my thoughts about pain.	-0.65	4031	0.03	1.21	1.20

*Negative item thresholds represent items that are easier to endorse, and positive thresholds represent items that are more difficult to endorse. SE, standard error of measure; score, raw score out of 6,048 (possible score of 7 × 864 people); Threshold, item endorsability threshold (expressed in logits); infit and outfit expressed as mean square (chi-square-based fit statistic).*

Item 2, the most readily endorsed item on the positive subscale, and item 20, the hardest to endorse item on the negative subscale demonstrated some underfit but all other items functioned well. Visual inspection of the PCA correlation matrix of the positive subscale indicated items 1 and 2 were clustered away from the other items and their residuals were marginally related (*r* = 0.30). No other meaningful patterns were detected and no meaningful patterns were detected in the negative subscale matrix, suggesting the subscales can be considered unidimensional. The positive and negative subscales remained reliable, with person separation indices of 0.86 and 0.87, respectively. Analysis of DIF revealed no meaningful biases.

#### Temporal Stability

A sub-sample of 172 participants answered the PMQ twice, 1 week apart. Both the positive and negative subscales of the PMQ demonstrated good test–retest reliability, with correlations of *r* = 0.76 (*p* < 0.001) and *r* = 0.72 (*p* < 0.001), respectively. Participants’ overall symptom changes on the PGIC were not correlated with 1-week changes on the PMQ positive subscale (*r* = 0.04, *p* = 0.58) or PMQ negative subscale (*r* = 0.01, *p* = 0.95), suggesting PMQ variation over a week is not associated with overall changes in participants’ pain.

#### Construct Validity

Data screening showed no violations of normality, linearity, homoscedasticity, multicollinearity, or other assumptions required for correlation and regression analyses. Correlations between the validation measures described above and the two subscales of the PMQ are shown Table 4. Providing evidence of convergent validity, both subscales significantly positively correlated with measures of pain intensity, pain interference, PC, pain cognitive intrusion, fear of pain, depression, anxiety, and metacognition, as expected. These were mostly significant at a conservative alpha level of *p* < 0.001. As expected, the negative PMQ subscale was significantly negatively correlated with mindfulness. Contrary to predictions, the negative correlation between mindfulness and the positive PMQ subscale did not reach significance. Providing evidence of discriminant validity, the positive PMQ subscale did not correlate with *a priori* unrelated demographic variables: country (*r* = 0.05, *p* = 0.14), marital status (*r* = 0.01, *p* = 0.77), and education level (*r* = -0.02, *p* = 0.66). The negative PMQ scale was also uncorrelated with country (*r* = 0.04, *p* = 0.22) and marital status (*r* = 0.02, *p* = 0.53), although it was very weakly associated with education (*r* = 0.07, *p* = 0.04).

**Table 4:** Correlations between the Pain Metacognitions Questionnaire and validation measures in initial validation sample (*N* = 864).

Variable	1	2	3	4	5	6	7	8	9	10
(1) Positive pain metacognitions (PMQ-P)	-									
(2) Negative pain metacognitions (PMQ-N)	0.16^***^	-								
(3) Pain intensity (BPI-P)	0.10^**^	0.16^***^	-							
(4) Pain interference (BPI-I)	0.14^***^	0.29^***^	0.68^***^	-						
(5) Pain catastrophizing (PCS)	0.28^***^	0.48^***^	0.45^***^	0.59^***^	-					
(6) Pain intrusion (ECIP)	0.25^***^	0.49^***^	0.32^***^	0.46^***^	0.70^***^	-				
(7) Pain-related fear (TSK)	0.36^***^	0.32^***^	0.24^***^	0.41^***^	0.49^***^	0.44^***^	-			
(8) Depression (HADS-D)	0.10^**^	0.34^***^	0.32^***^	0.56^***^	0.50^***^	0.49^***^	0.40^***^	-		
(9) Anxiety (HADS-A)	0.16^***^	0.42^***^	0.24^***^	0.40^***^	0.50^***^	0.48^***^	0.32^***^	0.59^***^	-	
(10) Metacognition (MCQ-30)	0.32^***^	0.39^***^	0.08^*^	0.19^**^	0.42^***^	0.43^***^	0.32^***^	0.41^***^	0.61^***^	-
(11) Mindfulness (MAAS)	-0.06	-0.26^**^	-0.01	-0.13^***^	-0.15^***^	-0.23^***^	-0.12^***^	-0.31^**^	-0.44^**^	-0.46^**^

*^∗^p < 0.05 (two-tailed), ^∗∗^p < 0.01 (two-tailed), ^∗∗∗^p < 0.001.*

As shown in Table 5, pain catastrophizing was significantly correlated with all subscales of the MCQ-30 and PMQ, and most strongly associated with the negative subscale of the PMQ. Furthermore, the PMQ explained unique variance in pain catastrophizing, accounting for a further 11 percent of variance in the PCS after controlling for the MCQ-30 (Table 6).

**Table 5:** Correlations between the Pain Metacognitions Questionnaire (PMQ), Pain Catastrophizing Scale (PCS), and subscales of the Metacognitions Questionnaire (MCQ-30) in initial validation sample (*N* = 864).

Variable	1	2	3	4	5	6	7
(1) Positive pain metacognitions (PMQ-P)	–						
(2) Negative pain metacognitions (PMQ-N)	0.16^∗∗∗^	–					
(3) Positive worry beliefs (MCQ-POS)	0.41^∗∗∗^	0.11^∗∗^	–				
(4) Negative worry beliefs (MCQ-NEG)	0.13^∗∗∗^	0.47^∗∗∗^	0.36^∗∗∗^	–			
(5) Cognitive confidence (MCQ-CC)	0.08^∗^	0.25^∗∗∗^	0.23^∗∗∗^	0.39^∗∗∗^	–		
(6) Need for control (MCQ-NC)	0.30^∗∗∗^	0.37^∗∗∗^	0.44^∗∗∗^	0.49^∗∗∗^	0.27^∗∗∗^	–	
(7) Cognitive self-consciousness (MCQ-CSC)	0.23^∗∗∗^	0.14^∗∗∗^	0.40^∗∗∗^	0.38^∗∗∗^	0.08^∗^	0.43^∗∗∗^	–
(8) Pain catastrophizing (PCS)	0.28^∗∗∗^	0.48^∗∗∗^	0.17^∗∗∗^	0.43^∗∗∗^	0.24^∗∗∗^	0.36^∗∗∗^	0.22^∗∗∗^

*^∗^p < 0.05 (two-tailed), ^∗∗^p < 0.01 (two-tailed), ^∗∗∗^p < 0.001.*

**Table 6:** Hierarchical regression analysis showing the unique contribution of the Pain Metacognitions Questionnaire (PMQ) to predicting the Pain Catastrophizing Scale after controlling for the Metacognitions Questionnaire (MCQ-30) (*N* = 864).

Predictor variables	*R*^2^	Δ*R*^2^	Δ*F*	Std. beta
**Step 1**	0.22	0.22	*F*(5,858) = 48.86^∗∗∗^	
MCQ-POS				-0.06
MCQ-NEG				0.31^***^
MCQ-CC				0.08^*^
MCQ-NC				0.20^***^
MCQ-CSC				0.04
**Step 2**	0.33	0.11	*F*(2,856) = 69.11^∗∗∗^	
MCQ-POS				-0.09^*^
MCQ-NEG				0.21^***^
MCQ-CC				0.06
MCQ-NC				0.09^*^
MCQ-CSC				0.05
PMQ-P				0.19^***^
PMQ-N				0.30^***^

*^∗^p < 0.05 (two-tailed), ^∗∗∗^p < 0.001 (two-tailed). MCQ-POS, positive beliefs about worry; MCQ-NEG, negative beliefs about uncontrollability and danger of worry; MCQ-CC, cognitive confidence; MCQ-NC, need for control; MCQ-CSC, cognitive self-consciousness; PMQ-P, positive pain metacognitions; PMQ-N, negative pain metacognitions.*

## Phase 2: Further Scale Validation

### Objectives

Phase 2 aimed to further validate the revised PMQ in a new sample to confirm that the scale functioned as expected when presented to participants as a 21-item scale on a 4-point Likert rating scale, rather than a 40-item scale on a 7-point Likert scale as initially tested.

### Methods

Phase 2 aimed to further validate the revised PMQ in a new sample to confirm that the scale functioned as expected when presented to participants as a 21-item scale on a 4-point Likert rating scale, rather than a 40-item scale on a 7-point Likert scale as initially tested.

#### Participants

A sample of 510 people was recruited online through the same Amazon Mechanical Turk (MTurk) forum described earlier. The same inclusion and exclusion criteria applied, except that only people with self-reported chronic pain were included this time. The survey was set up to exclude MTurk workers who had participated in the first validation survey.

#### Measures

Demographic questions and the following measures described in Phase 1 were used again: Brief Pain Inventory (BPI), Pain Catastrophizing Scale (PCS), Experience of Cognitive Intrusion in Pain (ECIP), and Hospital Anxiety Depression Scale (HADS). In addition, the Perseverative Thinking Questionnaire (PTQ) was administered to provide a more transdiagnostic measure of repetitive negative thinking than the ECIP. The revised 21-item Pain Metacognitions Questionnaire (PMQ) was also administered, scored on a 4-point Likert scale from 0 to 3 (see Supplementary Materials for final scale).

##### Perseverative thinking questionnaire

The Perseverative Thinking Questionnaire (PTQ) (Ehring et al., 2011) is a 15-item scale assessing various aspects of rumination and worry, including three subscales: core characteristics (e.g., “The same thoughts keep going through my mind again and again”), unproductiveness (e.g., “I keep asking myself questions without finding an answer”), and capturing mental capacity (e.g., “My thoughts prevent me from focusing on other things”). Using a 4-point Likert scale, PTQ scores range from 0 to 60 with higher scores indicating worse rumination. The PTQ has been validated in numerous samples, with high reliability of Cronbach’s α = 0.95 for the full scale and subscales ranging from α = 0.77, α = 0.94 (Ehring et al., 2011). The PTQ was used to establish convergent validity.

#### Procedure

The same procedures used to recruit the MTurk sample in Phase 1 were repeated, with data collected online using Qualtrics^TM^. Since retest reliability was not sought, the measures were only completed once by each participant.

#### Analysis

##### Rasch analysis of item functioning

The same Rasch analysis procedures described in Phase 1 were repeated to validate the findings of the initial analysis.

##### Construct validity

The same procedures used to assess convergent and discriminant validity in Phase 1 were repeated, however, with the slightly different battery of measures described above. Criterion validity was also sought by testing whether the PMQ was able to predict clinical levels of PC according to data showing a score of 24 on the PCS best identifies ‘patient’ status (Scott et al., 2014). The sample was dichotomised into those above and below this cut-off. A one-way analysis of variance was then conducted in SPSS to test whether there was a difference between high and low catastrophizing groups on PMQ scores for both subscales.

Further evidence of construct validity was sought using multiple hierarchical regression to test whether the PMQ would uniquely predict PC once other related variables were controlled. Predictor variables were entered into a SPSS hierarchical regression equation in five blocks: (1) demographics (age, gender, marital status, compensation status, work status, education, and pain duration); (2) pain intensity and interference (BPI); (3) emotional distress (HADS); (4) cognitive intrusion (ECIP) and perseverative thinking (PTQ); (5) pain metacognition (PMQ).

##### Identification of cut-off scores

Receiver operating characteristic (ROC) curve analysis was used to estimate preliminary cut-off thresholds for clinically meaningful scores on the two PMQ sub-scales based on their ability to predict clinical levels of PC. This was defined as scores of 24 and above on the PCS (Scott et al., 2014). ROC curves are commonly used to analyze and visualize the ability of screening tests to accurately predict dichotomous conditions, such as diagnostic status, by plotting a test’s sensitivity against 1-sensitivity (Swets, 1988; Zweig and Campbell, 1993). The area under the ROC curve (AUC) varies between 0.5 (depicting a test that is no better than chance at identifying the disorder), and 1.0 (depicting a perfect test that has 100% sensitivity and 100% specificity) (Hanley and McNeil, 1982). The AUC was calculated in MedCalc for Windows, version 17.7.2 (MedCalc Software, Ostend, Belgium). Following convention, a *p*-value < 0.05 for the AUC indicates it is significantly different from chance (0.5) and the test can therefore distinguish between cases and non-cases (Zhou et al., 2011).

Calculating cut-off scores involves balancing sensitivity and specificity. A common method that gives equal weight to sensitivity and specificity involves finding the point on the ROC curve that has the maximum vertical distance from the diagonal chance line, which is termed the Youden index (*J*) (Youden, 1950). Depending on the risks associated with false positives and false negatives, sensitivity or specificity can also be prioritized. Since the clinical application of the PMQ would likely involve further assessment through clinical interview, it was deemed more important to flag possible cases rather than avoid over-diagnosis. Therefore, sensitivity was prioritized over specificity, with cut-offs selected based on a sensitivity closest to 80%. Separate ROC analyses were performed for each PMQ subscale.

### Results

#### Sample Characteristics

Nearly all the 510 participants included in this sample lived in the United States (*n* = 496, 97.3%), although another six countries were represented. Demographic characteristics of the sample are shown in Supplementary Table S4, with overall very similar characteristics to the initial validation sample. Most participants were female (*n* = 306, 60%) and the mean age was 37.5 years (*SD* = 12.4). The mean pain duration was 6.43 years (*SD* = 7.44). A large proportion were employed (*n* = 362, 71%) and most were not involved in compensation claims (*n* = 449, 88%). The most common site of pain (see Supplementary Table S5) was the lower back (*n* = 305, 59.8%). Mean scores of the sample on the pain and psychological outcomes described above are presented in Table 7, with similar symptom levels as observed in the initial validation sample.

**Table 7:** Means, standard deviations and Cronbach’s coefficient alphas of outcome measures for second validation sample (*N* = 510).

Outcome	Mean	*SD*	Range	Interpretation	α
Positive pain metacognitions (PMQ-P)	-0.73	1.98	-6.45-6.15	–	0.88
Negative pain metacognitions (PMQ-N)	0.29	1.51	-6.01-6.3	–	0.87
Pain intensity (BPI-P)	4.97	1.66	0.25-10	Moderate (Anderson, 2005)	0.80
Pain interference (BPI-I)	4.73	2.47	0-10	–	0.92
Pain catastrophizing (PCS)	26.27	10.75	0-52	Clinical (Scott et al., 2014)	0.93
Cognitive intrusion of pain (ECIP)	27.75	15.33	0-60	–	0.97
Perseverative thinking (PTQ)	29.60	7.61	0-60	–	0.96
Depression (HADS-D)	6.72	4.29	0-21	Sub-clinical (Bjelland et al., 2002)	0.82
Anxiety (HADS-A)	9.09	4.66	0-21	Clinical (Bjelland et al., 2002)	0.82

*PMQ scores are expressed in logits rather than raw scores.*

#### Rasch Analysis of Item Functioning

Rasch analysis was conducted using the data from 510 people. Table 8 shows the average item endorsability thresholds and fit statistics for each subscale. Replicating the previous analysis, the positive subscale items were relatively hard to endorse, with mean (SD) person agreeability -0.72 (1.56) logits (range = -5.02 to 3.29 logits) less than the mean item endorsability 0 (0.74) logits (range = -1.56 to 0.84 logits). The negative subscale items were comparable with mean person agreeability 0.20 (1.28) logits (range = -4.74 to 5.15 logits) and mean item endorsability 0 (0.60) logits (range = -0.86 to 1.03 logits). Fifteen people (3%) registered a minimum score on the positive subscale and six people (<1%) registered a maximum score. Two (<1%) people registered a minimum score on the negative subscale and seven (1%) people registered a maximum score. These findings supported the suggestion that floor and ceiling effects are negligible.

**Table 8:** Categorical order, item endorsability thresholds and fit statistics for the revised 21-item version of the Pain Metacognitions Questionnaire (*N* = 510).

Positive Metacognitions Subscale						
**Item**		**Threshold**	**Score**	***SE***	**Infit**	**Outfit**

12	My pain won’t sneak up on me as long as I keep thinking about it.	0.84	505	0.08	1.04	1.06
8	My pain would get worse if I didn’t think about it a lot.	0.75	519	0.08	0.90	0.88
11	Focusing on the bad things about my pain helps me to enjoy the good things more.	0.73	523	0.08	1.08	1.07
5	Thinking a lot about my pain protects me from getting injured.	0.25	602	0.08	0.93	0.92
13	Thinking a lot about my pain helps me to cope with it.	0.22	607	0.08	0.85	0.87
16	Thinking about my pain helps me to understand myself better.	-0.14	668	0.08	0.94	0.94
1	My pain won’t improve unless I analyze it.	-0.43	715	0.08	1.03	1.04
10	Analyzing my pain prepares me for the worst.	-0.67	755	0.08	0.95	0.97
2	When I’m thinking about my pain I’m trying to problem solve it.	-1.56	900	0.08	1.25	1.24
**Negative Metacognitions Subscale**					
20	I don’t try to stop thinking about my pain because my thoughts seem to have a life of their own.	1.03	632	0.07	1.26	1.35
18	When I start thinking about my pain, it’s impossible to stop.	0.69	703	0.07	0.97	0.97
26	I make my pain worse by analyzing it.	0.56	729	0.07	1.00	1.03
39	I get caught in a vicious cycle of thinking about my pain and then thinking about how I wish I co…	0.43	756	0.07	0.90	0.90
40	When I find myself brooding on my pain, it starts me thinking about how I’m just making things worse.	0.19	803	0.07	0.82	0.82
38	I worry about the negative effects of thinking too much about my pain.	0.04	832	0.07	0.94	0.95
21	Thinking about my pain all the time makes me feel depressed.	0.03	834	0.07	1.26	1.23
33	I must block out my thoughts about pain.	-0.04	847	0.07	1.07	1.08
24	I would be less anxious if I didn’t focus on my pain as much.	-0.5	932	0.07	0.96	0.96
23	I feel stressed if I think a lot about my pain.	-0.78	981	0.08	0.84	0.81
22	I’d be happier if I stopped thinking about pain.	-0.79	984	0.08	0.93	0.91
36	It’s important to control my thoughts about pain.	-0.86	996	0.08	0.95	0.97

*Negative item thresholds represent items that are easier to endorse, and positive thresholds represent items that are more difficult to endorse. SE, standard error of measure; score, raw score out of 1,530 (possible score of 3 × 510 people); Threshold, item endorsability threshold (expressed in logits); infit and outfit expressed as mean square (chi-square-based fit statistic). Item numbers refer to the original 40-item draft version.*

Item 20, the hardest to endorse item on the negative subscale, demonstrated slight underfit but all other items functioned well. Visual inspection of the PCA correlation matrices for each of the subscales revealed no meaningful patterns in the data and contrast eigenvalues of 1.8 and 1.9 for the positive and negative subscales, respectively, suggested unidimensionality. No evidence of local dependence was noted suggesting no redundancy in the items.

Internal consistency reliability was maintained with person separation indices of 0.86 and 0.87 replicated for the positive and negative metacognitions subscales, respectively. Analysis of DIF revealed no meaningful biases. Overall, these findings support the refinement of the draft items to form two subscale measures of positive and negative metacognitions.

#### Construct Validity

Data screening showed no violations of normality, linearity, homoscedasticity, multicollinearity, or other assumptions required for correlation and regression analyses. Correlations between the validation measures described above and the two subscales of the PMQ are shown in Table 9. Providing evidence of convergent validity, both subscales significantly positively correlated with measures of pain intensity, pain interference, PC, pain cognitive intrusion, perseverative thinking, depression and anxiety, as expected, although not so strongly as to suggest scale redundancy (*r* = 0.1–0.56). These were mostly significant at a conservative alpha level of *p* < 0.001. Providing evidence of discriminant validity, the positive PMQ subscale did not correlate with *a priori* unrelated demographic variables: country (*r* = 0.02, *p* = 0.62), marital status (*r* = -0.06, *p* = 0.18), and education level (*r* = 0.07, *p* = 0.12). The negative PMQ scale was also uncorrelated with country (*r* = 0.05, *p* = 0.30) and marital status (*r* = -0.04, *p* = 0.39), although it was very weakly associated with education (*r* = -0.01, *p* = 0.86).

**Table 9:** Correlations between the Pain Metacognition Questionnaire and validation measures in further validation sample (*N* = 510).

Variable	1	2	3	4	5	6	7	8
(1) Positive pain metacognitions (PMQ-P)	–							
(2) Negative pain metacognitions (PMQ-N)	0.35^∗∗∗^	–						
(3) Pain intensity (BPI-P)	0.23^∗∗^	0.26^∗∗∗^	–					
(4) Pain interference (BPI-I)	0.19^∗∗∗^	0.42^∗∗∗^	0.66^∗∗∗^	–				
(5) Pain catastrophizing (PCS)	0.35^∗∗∗^	0.56^∗∗∗^	0.41^∗∗∗^	0.55^∗∗∗^	–			
(6) Pain intrusion (ECIP)	0.24^∗∗∗^	0.51^∗∗∗^	0.38^∗∗∗^	0.62^∗∗∗^	0.67^∗∗∗^	–		
(7) Perseverative thinking (PTQ)	0.21^∗∗∗^	0.52^∗∗∗^	0.19^∗∗∗^	0.37^∗∗∗^	0.50^∗∗∗^	0.63^∗∗∗^	–	
(8) Depression (HADS-D)	0.10^∗^	0.34^∗∗∗^	0.35^∗∗∗^	0.62^∗∗∗^	0.41^∗∗∗^	0.50^∗∗∗^	0.48^∗∗∗^	–
(9) Anxiety (HADS-A)	0.19^∗∗∗^	0.45^∗∗∗^	0.27^∗∗∗^	0.51^∗∗∗^	0.51^∗∗∗^	0.58^∗∗∗^	0.68^∗∗∗^	0.64^∗∗∗^

*^∗^p < 0.05 (two-tailed), ^∗∗^p < 0.01 (two-tailed), ^∗∗∗^p < 0.001 (two-tailed).*

Providing evidence of criterion validity, the PMQ predicted PC ‘patient’ status (Scott et al., 2014). For positive metacognitions, significant ANOVA results [*F*(1,508) = 35.22, *p* < 0.001] showed people with high PC scored higher on the PMQ (*M* = -0.31, *SD* = 1.97) than people with low PC (*M* = -1.33, *SD* = 1.83). For negative metacognitions, significant ANOVA results [*F*(1,508) = 89.62, *p* < 0.001] showed people with elevated PC scored higher on the PMQ (*M* = 0.78, *SD* = 1.51) than people with low PC (*M* = -0.41, *SD* = 1.21).

Further evidence of construct validity was provided by the ability of pain metacognition to predict unique variance in PC. As shown in Table 10, hierarchical multiple regression showed that the PMQ predicted a further 5% of PC when a range of demographic, pain and psychological variables were controlled (*R*^2^ change = 0.05, *p* < 0.001).

**Table 10:** Hierarchical regression analysis showing the unique contribution of pain metacognition to predicting pain catastrophizing (*N* = 510).

Predictor variables	*R*^2^	Δ*R*^2^	Δ*F*	Beta
**Step 1**	0.05	0.05	*F*(7,502) = 3.64^∗∗^	
Age				-0.01
Gender				0.51
Marital status				-0.32
Compensation				-0.28
Work status				0.15
Education				-0.89^**^
Pain duration				-0.07
**Step 2**	0.32	0.28	*F*(2,500) = 102.00^∗∗∗^	
Pain intensity (BPI-P)				0.80^*^
Pain interference (BPI-I)				0.33
**Step 3**	0.40	0.08	*F*(2,498) = 31.34^∗∗∗^	
Depression (HADS-D)				-0.05
Anxiety (HADS-A)				0.29^*^
**Step 4**	0.52	0.12	*F*(2,496) = 63.79^∗∗∗^	
Perseverative thinking (PTQ)				0.00
Cognitive intrusion (ECIP)				0.26^***^
**Step 5**	0.57	0.05	*F*(2,494) = 28.31^∗∗∗^	
Positive metacognition (PMQ-P)				0.70^***^
Negative metacognition (PMQ-N)				1.46^***^

*^∗^p < 0.05 (two-tailed), ^∗∗^p < 0.01 (two-tailed), ^∗∗∗^p < 0.001 (two-tailed).*

#### Identification of Cut-Off Scores

**FIGURE 2:**
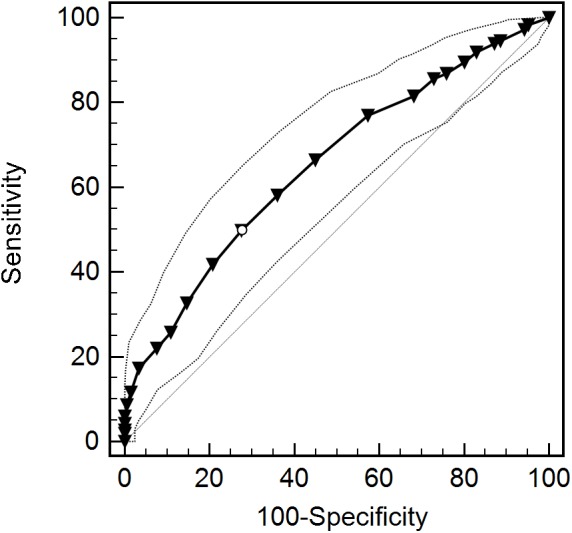
Receiver operating characteristic (ROC) curve and 95% CI for positive subscale of the Pain Metacognitions Questionnaire (PMQ-P).

The ROC curve analyses yielded significant AUC for both PMQ subscales, showing people with clinical levels of PC had higher positive metacognitions than those with non-clinical catastrophizing 65% of the time (95% CI 0.61–0.70, *SE* = 0.02, *p* < 0.0001). Similarly, people with clinical levels of PC had higher negative metacognitions than those with non-clinical PC 74% percent of the time (95% CI 0.70–0.78, *SE* = 0.02, *p* < 0.0001). The Youden index suggested a cut-off for the positive metacognitions subscale of >12 (*J* = 0.22, sensitivity = 49.8%, specificity = 72.5%), as depicted by a white circle on the ROC curve in Figure 2. However, prioritizing sensitivity over specificity suggests a PMQ-P score of >9 (sensitivity = 76.9%, specificity = 42.6%), provides a better cut-off, as shown in Table 11.

**Table 11:** Coordinates of the ROC curve for values of the Pain Metacognitions Questionnaire, positive subscale (PMQ-P).

Score (PMQ-P)	Sensitivity	95% CI	Specificity	95% CI
>0	98.33	96.1-99.5	4.74	2.3-8.5
>2	94.65	91.5-96.9	11.37	7.4-16.5
>5	89.63	85.6-92.8	19.91	14.7-25.9
>7	85.62	81.1-89.4	27.01	21.1-33.5
>8	81.61	76.7-85.8	31.75	25.5-38.5
>9	76.92	71.7-81.6	42.65	35.9-49.6
>12	49.83	44.0-55.6	72.51	66.0-78.4

*Clinical levels of pain catastrophizing (PCS > 24), n = 299; non-clinical catastrophizing, n = 211.*

**FIGURE 3:**
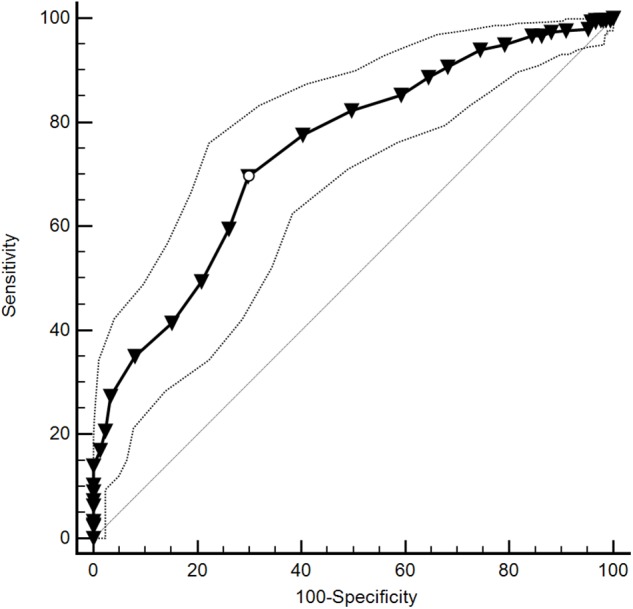
Receiver operating characteristic (ROC) curve and 95% CI for negative subscale of the Pain Metacognitions Questionnaire (PMQ-N).

For the negative metacognitions subscale, the Youden index suggested a cut-off of >19 (*J* = 0.40, sensitivity = 69.6%, specificity = 70.1%), as depicted by a white circle on the ROC curve in Figure 3. However, prioritizing sensitivity over specificity suggests a PMQ-N score of >18 (sensitivity = 77.6%, specificity = 59.7%) provides a better cut-off, as shown in Table 12.

**Table 12:** Coordinates of the ROC curve for values of the Pain Metacognitions Questionnaire, negative subscale (PMQ-N).

Score (PMQ-N)	Sensitivity	95% CI	Specificity	95% CI
>0	99.67	98.2-100.0	0.47	0.01-2.6
>12	94.98	91.9-97.2	20.85	15.6-27.0
>14	90.64	86.8-93.7	31.75	25.5-38.5
>16	85.28	80.8-89.1	40.76	34.1-47.7
>17	82.27	77.5-86.4	50.24	43.3-57.2
>18	77.59	72.4-82.2	59.72	52.8-66.4
>19	69.57	64.0-74.7	70.14	63.5-76.2

## Discussion

This study aimed to develop a psychometrically sound self-report measure of unhelpful metacognitions underlying pain-related rumination, thereby operationalizing a new variable to target in pain research and treatment. Worry and rumination are key psychological processes underlying pain catastrophizing, which is strongly linked to negative pain and health outcomes (Quartana et al., 2009). Metacognitions, or beliefs about thinking, have been shown to drive worry and rumination in people with anxiety and mood disorders (Fisher and Wells, 2009), suggesting they may underlie similar processes in people with pain. However, despite the existence of a generic self-report measure of metacognition, (Wells and Cartwright-Hatton, 2004) there had been no instrument tailored to the pain experience that also encompassed beliefs pertaining to both anxiety-related worry and depressogenic rumination. This study therefore filled this research-practice gap, showing it is possible to reliably measure pain metacognitions with a self-report instrument.

The resulting Pain Metacognitions Questionnaire (PMQ) is a 21-item bi-dimensional scale comprising a 9-item positive metacognition subscale (PMQ-P) measuring the extent to which people believe thinking a lot about their pain is helpful, and a 12-item negative metacognition subscale (PMQ-N) measuring how uncontrollable and damaging people believe their thinking about pain to be. Both types of metacognition are unhelpful due to their tendency to facilitate worry and rumination. The scales have good internal consistency reliability (PSI = 0.86, 0.87, respectively) and test–retest reliability (*r* = 0.76, 0.72, respectively), as well as minimal floor and ceiling effects, making them suitable for use in both research and clinical settings. As predicted, they also positively correlate with measures of pain intensity, disability, PC, perseverative thinking, cognitive intrusion of pain, fear of pain, depression, anxiety, metacognition, and negatively correlate with mindfulness. These correlations are mostly of moderate strength, providing good evidence of construct validity but not redundancy. Based on its ability to predict clinical levels of PC, a PMQ-P score > 9 or PMQ-N score > 18 is likely to be clinically meaningful, although it should be noted that these values are associated with relatively low specificity so further qualitative assessment is recommended at the clinical level. Importantly, the PMQ explains unique variance in pain catastrophizing when controlling for the MCQ-30, suggesting this new pain-specific measure is better at explaining PC than the generic MCQ. Such incremental utility has been highlighted as an important criterion for adopting new measures of metacognition (Bailey and Wells, 2015).

The two-dimensional nature of the PMQ is consistent with metacognitive theory, which describes attitudes toward cognition as falling into distinct positive and negative categories (Wells and Matthews, 1996; Fisher and Wells, 2009). It also reflects previously published qualitative results (Schütze et al., 2017). That these qualitative interviews were informed by metacognitive theory probably influenced this dimensionality, in that participants were asked about their perceptions of the positive and negative aspects of rumination/worry about pain. The PMQ’s dimensionality also mirrors the factor structure of a similar disorder-specific self-report measure of metacognition for people with chronic fatigue syndrome (Fernie et al., 2015). Although the PMQ’s bi-dimensionality rules out the convenience of a single full-scale score, it may be helpful in future clinical applications of the scale. For example, metacognitive therapy (MCT), although so far untested in people with chronic pain, targets positive and negative metacognitions in different ways (Wells, 2009), making the structure of the PMQ useful in any future trials of MCT for people with pain.

Rasch analysis showed that participants found it harder to endorse positive metacognitions than negative ones. This is also consistent with MCT literature showing that in clinical settings it is often harder to elicit positive metacognitions. Negative metacognitions are therefore targeted first in MCT to allow time for meta-cognitive awareness to develop to a sufficient level for positive beliefs about worry/rumination to become more recognizable to the individual (Wells, 2009). Considering this, it is unsurprising that people found it somewhat difficult to endorse positive metacognitions with a self-report questionnaire. The PMQ was nevertheless able to assess these beliefs with minimal floor effects, suggesting it remains a valid measure of positive metacognitions.

Many of the metacognitions reflected in the PMQ also echo existing theoretical models of pain-related worry. For example, item 2 of the final scale (see Supplementary Materials) describes thinking about pain as a form of problem-solving. This is consistent with the misdirected problem solving model of pain-related worry, which sees worry as an attempt to solve the problem of how to relieve persistent pain (Eccleston and Crombez, 2007). Items 1 and 4 are also consistent with this model in that they describe thinking about pain as a strategy to resolve pain or prevent it from getting worse. Similarly, items 6, 8, and 9 characterize worry/rumination as a form of coping, which is consistent with research depicting PC as a coping behavior (Rosenstiel and Keefe, 1983), as well as more recent functional analytic accounts of worry about pain as a self-regulation strategy aimed at reducing emotional distress (Flink et al., 2013). Item 3 depicting worry/rumination as a strategy to prevent injury is consistent with a ‘commonsense model’ of pain-related fear, linking avoidance behavior to representations of pain as a sign of structural damage (Bunzli et al., 2017). Similarly, the uncontrollability of worry/rumination captured by items 10 and 11 (items on the negative subscale) is consistent with models of hypervigilance (van Damme et al., 2004) and cognitive intrusion (Attridge et al., 2015), which depict pain-related stimuli, including thoughts, as difficult to disengage from.

The research reported here has several strengths, including that it used a rigorous evidence-informed approach to scale development. While many self-report measures are drafted based on theory and expert opinion (Streiner and Norman, 2008), items for the PMQ emerged out of rich qualitative data as well as theory. Secondly, the two validation studies employed large samples that were well powered and largely comprised people with chronic pain rather than being non-clinical cohorts. Thirdly, the psychometric evaluation of PMQ data employed rigorous Rasch analysis based on item response theory, which has several advantages over classical test theory techniques such as factor analysis. For example, IRT allows for a more thorough analysis of individual item functioning as well as producing true interval-level scales rather than ordinal scales produced in CTT (DeVellis, 2012). The IRT techniques used here also allowed for a data-driven approach to optimizing response format, with the final 4-point Likert scale derived from an analysis of how participants used the scale. Finally, this study involved two phases or validation, with the final version of the scale tested in a fresh sample rather than relying on a single sample as is common during initial validation of new scales using exploratory factor analysis.

However, this study is not without limitations. Firstly, PMQ items use the terms ‘thinking,’ ‘thinking a lot,’ and ‘analyzing’ as generic referents to repetitive negative thinking, based on scale piloting feedback that the term ‘rumination’ was not always clear. However, it is possible that these terms do not convey perseveration of thought as much as ‘worry’ and ‘rumination.’ Secondly, while the qualitative scale development sample (Schütze et al., 2017) was a pain clinic sample that is representative of many other clinical samples, the validation studies employed internet samples which had lower pain intensity and disability than many pain clinic samples (Tardif et al., 2016), despite their clinical levels of catastrophizing. This may limit generalizability of these results and highlights the need to validate the PMQ in other samples with chronic pain disorders, such as those found in treatment settings where pain diagnoses are assessed by health professionals rather than merely self-reported. Furthermore, these results are susceptible to the biases inherent in all studies using self-reported data, such as social desirability bias, recall bias and context effects (DeVellis, 2012).

Another significant limitation, which highlights the need for further research, is the fact that these three linked studies used cross-sectional designs, notwithstanding the test–retest analysis. This means it is unclear whether the PMQ is sensitive to treatment-related changes in pain metacognitions and therefore whether it can be used as an outcome measure in intervention research. Future validation studies could address this by collecting pre- and post-intervention data, particularly during psychological or multidisciplinary treatments that aim to reduce pain-related rumination and PC. An important question is whether changes in PC are associated with changes in pain metacognition (PMQ). Future research using prospective designs is needed to answer this. Prospective research is also needed to refine estimates of clinically meaningful cut-offs since the present suggestions should be considered preliminary and subject to revision.

More broadly, prospective research is needed to test the theoretical model that underlies this new measure, namely a metacognitive model of pain-related rumination. In metacognitive theory, unhelpful positive and negative metacognitions function as risk factors for subsequent worry/rumination and indeed evidence in psychopathology literature supports this (Papageorgiou and Wells, 2009). Future research should therefore test whether baseline PMQ scores predict future episodes of pain rumination or PC, as would be expected. A precursor to this could also be to test whether metacognition moderates the relationship between pain intensity and rumination/worry, as would be expected according to metacognitive theory. Future research could also use the new PMQ to replicate findings from previous pain metacognition studies that used the generic MCQ-30 (e.g., Yoshida et al., 2012; Spada et al., 2016; Ziadni et al., 2018). Lastly, an obvious avenue for future research based on this theoretical work is the development of interventions targeting metacognitions, such as a form of MCT for people with elevated PC. Present findings suggest this is warranted and that metacognition is a promising future treatment target.

In summary, this study shows that pain-related metacognitions can be validly and reliably measured using a new self-report instrument. The PMQ can be used in clinical and research settings and operationalizes a psychological variable that warrants further investigation as a potential new treatment target in pain research. This has the potential to improve the efficacy of interventions for outcomes such as pain catastrophizing and other forms of pain-related distress.

## Ethics Statement

This study was carried out in accordance with the recommendations of ‘NHMRC National Statement on Ethical Conduct in Human Research’ with online informed consent from all subjects. All subjects gave online informed consent in accordance with the Declaration of Helsinki. The protocol was approved by the ‘South Metropolitan Health Service Human Research Ethics Committee’.

## Author Contributions

RS, CR, AS, HS, and PO’S participated in study design and data interpretation. RS and MC participated in data analysis. RS conducted data collection. All authors were involved in manuscript preparation and editing.

## Conflict of Interest Statement

The authors declare that the research was conducted in the absence of any commercial or financial relationships that could be construed as a potential conflict of interest.

## Footnotes

1 http://psych.hanover.edu/research/exponnet.html

2 http://www.mturk.com

3 https://www.winsteps.com/index.htm
