# Supplementary material for: Assessing Beliefs Underlying Rumination About Pain: Development and Validation of the Pain Metacognitions Questionnaire
Source: Front Psychol. 2019 Apr 26;10:910. doi: 10.3389/fpsyg.2019.00910 (PMC6497779; doi:10.3389/fpsyg.2019.00910)
Supplement: Supplementary file 1 [file Table_1.DOCX]

Supplementary Material

Assessing beliefs underlying rumination about pain: Development and validation of the Pain Metacognitions Questionnaire

Robert Schütze^*^, Clare Rees, Anne Smith, Helen Slater, Mark Catley, and Peter O’Sullivan

*** Correspondence:** Robert Schütze: r.schutze@curtin.edu.au

Supplementary Table 1. Demographic characteristics of initial validation sample (N = 864)

| **Variable** | **Frequency** | **%** |
| --- | --- | --- |
| Gender  Male  Female | 279  585 | 32.3  67.7 |
| Marital status  Married  Defacto  Single  Widow | 409  89  356  10 | 47.3  10.3  41.2  1.2 |
| Compensation claim  No  Yes, workers  Yes, motor  Yes, other | 803  28  10  23 | 92.9  3.2  1.2  2.7 |
| Work status  Full-time paid  Part-time paid  Unemployed (pain-related)  Unemployed (not pain-related)  Leave (pain-related)  Studying  Retraining  Retired  Home duties  Voluntary work  Other | 397  145  81  32  8  68  2  34  39  2  56 | 45.9  16.8  9.4  3.7  .9  7.9  .2  3.9  4.5  .2  6.5 |
| Education  <10 years schooling  10 years schooling  12 years schooling  Apprenticeship/technical  University Bachelor degree  University postgraduate degree | 10  47  197  121  314  175 | 1.2  5.4  22.8  14.0  36.3  20.3 |

Supplementary Table 2. Frequency statistics for pain sites in initial validation sample (N=864)

| **Pain site** | **Frequency** | **%** |
| --- | --- | --- |
| Head/face | 41 | 4.7 |
| Neck | 187 | 21.6 |
| Upper limb (including shoulder, elbow, hands) | 181 | 20.9 |
| Chest | 39 | 4.5 |
| Upper back | 125 | 14.5 |
| Abdomen | 20 | 2.3 |
| Lower back | 347 | 40.2 |
| Lower limb (including, hip, knee, ankle) | 307 | 35.5 |
| Pelvis/genitals | 36 | 4.2 |
| Widespread pain (all over my body) | 126 | 14.6 |

*Note.* Participants could endorse more than one category.

Supplementary Table 3. Categorical order, item endorsability thresholds and fit statistics for the draft 40-item version of the Pain Metacognitions Questionnaire (N=864).

| Positive Metacognitions Subscale | | | | | | |
| --- | --- | --- | --- | --- | --- | --- |
| Item |  | Threshold | Score | SE | Infit | Outfit |
| 12 | My pain won’t sneak up on me as long as I keep thinking about it. | 0.56 | 2129 | 0.03 | 0.83 | 0.83 |
| 8 | My pain would get worse if I didn’t think about it a lot. | 0.54 | 2144 | 0.03 | 0.88 | 0.91 |
| 11 | Focusing on the bad things about my pain helps me to enjoy the good things more. | 0.44 | 2247 | 0.03 | 1.15 | 1.29 |
| 13 | Thinking a lot about my pain helps me to cope with it. | 0.27 | 2443 | 0.03 | 0.76 | 0.79 |
| 9 | I’m better prepared for pain if I think about it a lot. | 0.25 | 2464 | 0.03 | 0.71 | 0.70 |
| 15 | I feel more in control when I’m thinking about my pain. | 0.14 | 2600 | 0.03 | 0.75 | 0.79 |
| 7 | I won’t get injured as easily if I stay focused on my pain. | 0.12 | 2618 | 0.03 | 0.86 | 0.89 |
| 14 | I should stop thinking so much about my pain because it doesn’t help. | 0.03 | 2732 | 0.03 | 1.35 | 1.96 |
| 5 | Thinking a lot about my pain protects me from getting injured. | -0.04 | 2821 | 0.03 | 1.02 | 1.02 |
| 10 | Analyzing my pain prepares me for the worst. | -0.08 | 2880 | 0.03 | 0.82 | 0.82 |
| 16 | Thinking about my pain helps me to understand myself better. | -0.12 | 2922 | 0.03 | 0.86 | 0.90 |
| 4 | Thinking about pain doesn't get you anywhere. | -0.15 | 2959 | 0.03 | 1.29 | 1.43 |
| 1 | My pain won’t improve unless I analyze it. | -0.21 | 3045 | 0.03 | 1.18 | 1.18 |
| 6 | Thinking about my pain all the time means I’m more aware of my body so I’m less likely to hurt myself. | -0.26 | 3101 | 0.03 | 1.11 | 1.25 |
| 3 | Analyzing my pain will help me to find a solution and get better | -0.69 | 3649 | 0.03 | 1.19 | 1.19 |
| 2 | When I’m thinking about pain I’m trying to problem solve | -0.81 | 3786 | 0.03 | 1.35 | 1.37 |
| Negative Metacognitions Subscale | | | | | | |
| Item |  | Threshold | Score | SE | Infit | Outfit |
| 34 | When thoughts about my pain come to mind, I try to just get on with what I’m doing. | 0.8 | 2170 | 0.03 | 1.24 | 1.85 |
| 31 | My family suffers because I think about my pain so much. | 0.41 | 2717 | 0.03 | 0.99 | 0.97 |
| 20 | I don't try to stop thinking about my pain because my thoughts seem to have a life of their own. | 0.35 | 2811 | 0.03 | 1.09 | 1.17 |
| 29 | I’m no fun to be around because I’m so focused on pain. | 0.32 | 2861 | 0.03 | 0.94 | 0.93 |
| 18 | When I start thinking about my pain, it’s impossible to stop. | 0.28 | 2923 | 0.02 | 0.94 | 0.96 |
| 26 | I make my pain worse by analyzing it. | 0.21 | 3038 | 0.02 | 0.98 | 1.01 |
| 32 | If I could stop thinking about my pain, I would have better relationships. | 0.18 | 3084 | 0.02 | 0.86 | 0.87 |
| 30 | People would like me more if I focused less on my pain. | 0.15 | 3137 | 0.02 | 0.86 | 0.88 |
| 39 | I get caught in a vicious cycle of thinking about my pain and then thinking about how I wish I could stop thinking about it. | 0.15 | 3143 | 0.02 | 0.78 | 0.79 |
| 17 | I can’t help thinking about my pain all the time. | 0.14 | 3157 | 0.02 | 1.15 | 1.20 |
| 27 | It hurts more when I think about my pain too much. | 0.11 | 3206 | 0.02 | 1.16 | 1.20 |
| 25 | I have less pain when I don’t think about it so much. | 0.09 | 3231 | 0.02 | 1.33 | 1.39 |
| 33 | I must block out my thoughts about pain. | 0.04 | 3307 | 0.02 | 0.82 | 0.92 |
| 28 | My thoughts don’t affect my pain levels. | 0 | 3386 | 0.02 | 1.56 | 1.67 |
| 40 | When I find myself brooding on my pain, it starts me thinking about how I’m just making things worse. | -0.02 | 3409 | 0.02 | 0.71 | 0.72 |
| 19 | It's easy to shift my attention away from thoughts about pain. [R] | -0.03 | 3426 | 0.02 | 1.15 | 1.23 |
| 38 | I worry about the negative effects of thinking too much about my pain. | -0.15 | 3616 | 0.03 | 0.92 | 0.92 |
| 37 | When I realize I’m thinking too much about my pain, I get annoyed with myself. | -0.27 | 3799 | 0.03 | 0.88 | 0.87 |
| 24 | I would be less anxious if I didn't focus on my pain as much. | -0.31 | 3868 | 0.03 | 0.82 | 0.84 |
| 21 | Thinking about my pain all the time makes me feel depressed. | -0.34 | 3902 | 0.03 | 1.00 | 0.98 |
| 35 | When thoughts about my pain grab my attention, I try to push them out of my mind. | -0.47 | 4099 | 0.03 | 1.15 | 1.23 |
| 22 | I’d be happier if I stopped thinking about pain. | -0.5 | 4131 | 0.03 | 0.77 | 0.78 |
| 23 | I feel stressed if I think a lot about my pain. | -0.52 | 4168 | 0.03 | 0.87 | 0.85 |
| 36 | It’s important to control my thoughts about pain. | -0.62 | 4293 | 0.03 | 1.05 | 1.03 |

*Note.* Bold type shows excessive item misfit; negative item thresholds represent items that are easier to endorse, and positive thresholds represent items that are more difficult to endorse. SE=standard error of measure; score=raw score out of 6,048 (possible score of 7 × 864 people); Threshold =item endorsability threshold (expressed in logits); infit and outfit expressed as mean square (chi-square-based fit statistic).

Supplementary Table 4. Demographic characteristics of further validation sample (N = 510)

| **Variable** | **Frequency** | **%** |
| --- | --- | --- |
| Gender  Male  Female | 204  306 | 40.0  60.0 |
| Marital status  Married  Defacto  Single  Widow | 270  11  226  3 | 52.9  2.2  44.3  0.6 |
| Compensation claim  No  Yes, workers  Yes, motor  Yes, other | 449  36  10  15 | 88.0  7.1  2.0  2.9 |
| Work status  Full-time paid  Part-time paid  Unemployed (pain-related)  Unemployed (not pain-related)  Leave (pain-related)  Studying  Retired  Home duties  Voluntary work  Other | 292  70  29  19  4  16  20  40  4  16 | 57.3  13.7  5.7  3.7  .8  3.1  3.9  7.8  .8  3.1 |
| Education  <10 years schooling  10 years schooling  12 years schooling  Apprenticeship/technical  University Bachelor degree  University postgraduate degree | 2  7  134  90  208  69 | 0.4  1.4  26.3  17.6  40.8  13.5 |
| Pain frequency  Always present (intensity constant)  Always present (intensity varies)  Often present  Occasionally present  Rarely present | 25  229  133  95  28 | 4.9  44.9  26.1  18.6  5.5 |
| Pain diagnosis  Yes  No | 210  300 | 41.2  58.8 |
| Psychological diagnosis  Yes  No | 98  412 | 19.2  80.8 |

Supplementary Table 5. Frequency statistics for pain sites in further validation sample (N=510)

| **Pain site** | **Frequency** | **%** |
| --- | --- | --- |
| Head/face | 29 | 5.7 |
| Neck | 90 | 17.6 |
| Upper limb (including shoulder, elbow, hands) | 101 | 19.8 |
| Chest | 9 | 1.8 |
| Upper back | 73 | 14.3 |
| Abdomen | 20 | 3.9 |
| Lower back | 205 | 40.2 |
| Lower limb (including, hip, knee, ankle) | 156 | 30.6 |
| Pelvis/genitals | 12 | 2.4 |
| Widespread pain (all over my body) | 39 | 7.6 |

**Pain Metacognitions Questionnaire (PMQ)**

*We all think about pain in different ways. Please help us to understand your attitudes towards your own thoughts about pain. Rate how much you agree or disagree with the statements below. There are no right or wrong answers.*

0 = Strongly Disagree

1 = Disagree

2 = Agree

3 = Strongly Agree

| 1. | My pain won’t improve unless I analyse it. | 0 | 1 | 2 | 3 |
| --- | --- | --- | --- | --- | --- |
| 2. | When I’m thinking about my pain I’m trying to problem solve it. | 0 | 1 | 2 | 3 |
| 3. | Thinking a lot about my pain protects me from getting injured. | 0 | 1 | 2 | 3 |
| 4. | My pain would get worse if I didn’t think about it a lot. | 0 | 1 | 2 | 3 |
| 5. | Analyzing my pain prepares me for the worst. | 0 | 1 | 2 | 3 |
| 6. | Focusing on the bad things about my pain helps me to enjoy the good things more. | 0 | 1 | 2 | 3 |
| 7. | My pain won’t sneak up on me as long as I keep thinking about it. | 0 | 1 | 2 | 3 |
| 8. | Thinking a lot about my pain helps me to cope with it. | 0 | 1 | 2 | 3 |
| 9. | Thinking about my pain helps me to understand myself. | 0 | 1 | 2 | 3 |
| 10. | When I start thinking about my pain, it’s impossible to stop. | 0 | 1 | 2 | 3 |
| 11. | I don't try to stop thinking about my pain because my thoughts seem to have a life of their own. | 0 | 1 | 2 | 3 |
| 12. | Thinking about my pain all the time makes me feel depressed. | 0 | 1 | 2 | 3 |
| 13. | I’d be happier if I stopped thinking about pain. | 0 | 1 | 2 | 3 |
| 14. | I feel stressed if I think a lot about my pain. | 0 | 1 | 2 | 3 |
| 15. | I would be less anxious if I didn't focus on my pain as much. | 0 | 1 | 2 | 3 |
| 16. | I make my pain worse by analysing it. | 0 | 1 | 2 | 3 |
| 17. | I must block out my thoughts about pain. | 0 | 1 | 2 | 3 |
| 18. | It’s important to control my thoughts about pain. | 0 | 1 | 2 | 3 |
| 19. | I worry about the negative effects of thinking too much about my pain. | 0 | 1 | 2 | 3 |
| 20. | I get caught in a vicious cycle of thinking about my pain and then thinking about how I wish I could stop thinking about it. | 0 | 1 | 2 | 3 |
| 21. | When I find myself brooding on my pain, it starts me thinking about how I’m just making things worse. | 0 | 1 | 2 | 3 |

## PMQ Scoring Instructions:

Sum items 1-9 for positive metacognitions subscale score (PMQ-P);

Sum items 10-21 for negative metacognitions subscale score (PMQ-N).

(The PMQ has two dimensions and a single full-scale score is not supported by PMQ validation analysis.)

A PMQ-P score >9 or PMQ-N score >18 indicates greater likelihood of elevated pain catastrophizing, suggesting further assessment is indicated
